# Navigating Boundaries: How Pharmacists Develop Their Clinical Identity in a Complex Multidisciplinary Healthcare Setting

**DOI:** 10.5334/pme.1597

**Published:** 2025-05-07

**Authors:** Lucille Crafford, Malou Stoffels, Chrisna Wagenaar, Elmien Bronkhorst, Andries Gous, Rashmi A. Kusurkar, Anouk Wouters

**Affiliations:** 1 Department of Clinical Pharmacy, School of Pharmacy, Sefako Makgatho Health Sciences University, South Africa; 2 Amsterdam UMC location Vrije Universiteit Amsterdam, Research in Education, De Boelelaan 1118, Amsterdam, The Netherlands; 3 Amstel Academy, Amsterdam UMC, The Netherlands; 4 LEARN! research institute for learning and education, Faculty of Psychology and Education, VU University Amsterdam, The Netherlands; 5 Amsterdam Public Health, Quality of Care, Amsterdam, The Netherlands

## Abstract

**Introduction:**

Clinical pharmacists are crucial for optimizing medication therapy and improving patient outcomes, yet their potential is underutilized in many low- to middle-income countries. Shifting from traditional dispensing to clinical roles requires professional development and identity transformation. In South Africa’s public healthcare system, this shift faces additional challenges, such as a lack of formal positions, limited resources, and role ambiguity. Understanding how clinical pharmacists navigate this transition and develop their clinical identity is essential for their integration into healthcare teams and for improving patient care.

**Methods:**

Using a constructivist approach, this qualitative study employed semi-structured interviews with clinical pharmacists (n = 12) across South Africa’s public healthcare sector. We analyzed data through the lens of boundary crossing. Through thematic analysis we explored how pharmacists navigate the complexities of transitioning from dispensing to broader clinical roles, and how these experiences shape their professional identity.

**Results:**

Clinical pharmacists navigated both intrapersonal and interpersonal boundaries in their evolving roles. Three key themes were identified: (a) Bridging the gap within: Pharmacists navigate intrapersonal boundaries for clinical identity formation, (b) Bridging the gap between: Pharmacists navigate interprofessional boundaries for collaboration and identity formation, and (c) Building bridges: Pharmacists employ strategies to promote collaboration and recognition.

**Discussion:**

This study uncovered the complex interplay between intrapersonal boundaries – such as role ambiguity, self-doubt, reconciling traditional dispensing roles with expanded clinical responsibilities, and the need for mentorship – and interpersonal boundaries, including hierarchical structures, unclear role expectations, limited recognition, and challenges in interprofessional collaboration, in shaping clinical pharmacists’ identities. Fostering boundary crossing competence and interprofessional collaboration can help overcome systemic barriers, enabling pharmacists to navigate their roles, advocate for their expertise, and gain recognition within healthcare teams, ultimately enhancing their integration and improving patient care in resource-constrained settings like South Africa.

## Introduction

Clinical pharmacists are increasingly recognized as essential healthcare team members due to their role in optimizing medication therapy and improving patient outcomes [1,2,3]. Their contributions extend beyond dispensing to include comprehensive medication management, including preventing medication-related harm, and collaborating with other healthcare professionals [1,2,3,4,5,6]. However, despite growing recognition of their role, the full potential of clinical pharmacists in practice remains unrealized [7]. Studies from Brazil, Pakistan and China have indicated that implementing clinical pharmacy services in hospitals remains incomplete [8,9,10], which is also true for South Africa [2,7,11,12]. Transitioning from traditional dispensing to clinical pharmacy roles requires significant professional development and identity transformation [13,14,15]. Pharmacists must navigate the blurred boundaries between dispensing and clinical practice, negotiating new roles and expectations [13]. While this shift offers opportunities for comprehensive medication management [13,14,15], clinical pharmacists in resource-constrained settings like South Africa face numerous challenges, including a lack of formal positions and limited resources [2,7,11,16]. These issues are most profound in the public sector, which serves the majority of the population [7,12,17,18,19]. Additionally, a lack of mentorship and misunderstandings about the clinical pharmacist’s role hinder integration into healthcare teams and impact their identity formation and ultimately patient care [7,20,21,22,23,24,25].

Existing literature indicates that a strong professional identity is essential for confident practice and contributes to job satisfaction, and career commitment among healthcare professionals [26]. Professional identity is defined as “the attitudes, values, knowledge, beliefs, and skills shared with others within a professional group” [27], encompassing competencies, ethics, and both personal and group identity, which collectively shape individuals’ understanding of their professional roles [28]. Professional identity formation (also referred to as identity development) is a complex process involving the internalization of professional values, norms, and behaviors [29], shaped by personal experiences, cultural influences, and interactions with role models [26,29,30,31,32]. While identities are dynamic and evolving, they are foundational to how professionals perceive themselves and their role within healthcare teams [33].

Previous research on the professional identity formation of clinical pharmacists has mainly explored factors like educational experiences, workplace culture, and mentorship. These studies indicate that, despite recognizing the importance of a pharmacist-clinician identity, pharmacists often feel their clinical skills are underutilized [8,9,34,35]. Role modeling is crucial for identity formation [26,29,32], and its absence limits opportunities for identity matching and learning through socialization [13]. The unique challenges faced by clinical pharmacists in low-to middle income countries (LMICs), like South Africa [12], within a complex healthcare landscape, necessitate further investigation to understand how these factors specific to their context influence professional identity formation. Currently, in South Africa, clinical pharmacy cannot be registered as a specialization, resulting in the absence of official clinical pharmacy positions. Consequently, clinical pharmacists are rarely appointed to allocated posts in the public sector. Our previous study [11] found that those not in dedicated positions often experience a lack of motivation and support from other healthcare professionals, which could impede their ability to develop a strong professional identity and fully utilize their clinical skills.

This study investigates how clinical pharmacists in South Africa’s public healthcare system navigate professional identity formation, addressing the gap in understanding the complexities of their transition from dispensing to broader clinical roles. Using Boundary Crossing as a theoretical lens, it explores how pharmacists navigate “discontinuities” or “tensions” in these transitions and how these experiences shape their identity. Boundary crossing is a dynamic process involving learning and adaptation as individuals navigate discontinuities between professional domains [35]. Successfully navigating boundary experiences, including communication and collaboration across different practices, is essential for successful integration into interprofessional teams [36]. Boundary objects, like patient care plans or medication reconciliation tools, can facilitate collaboration, while boundary brokers, such as experienced clinical pharmacists, can support the transition process [35,37,38].

The value of the boundary-crossing perspective in studying professional formation lies in its recognition that boundaries hold learning potential. It is not just about overcoming obstacles but harnessing the potential of these boundaries as spaces for learning and innovation [33]. Attempts to link related yet disparate contexts create potential for professional identity formation through boundary crossing [35,38,39]. Engaging with different perspectives enables individuals to develop new skills, enriching their understanding of current practices and eventually transforming them for the better [35]. As this process involves both individual agency and organizational support, the individual’s perspective is always situated within their work context [40]. By understanding the interplay between intra- and interpersonal factors, including the influence of workplace culture, role models, and support systems [38,41], this study seeks to address the gap in knowledge regarding the experiences of clinical pharmacists in navigating this complex transition, focusing on professional identity formation. Understanding these challenges and facilitators is crucial for informing educational approaches that support clinical pharmacists’ development. By supporting clinical pharmacists in their transition to expanded roles, their impact on patient care can be optimized, fostering more confidence and commitment in the clinical pharmacists’ workforce.

### Research Question

How do clinical pharmacists develop their identities within a complex multidisciplinary healthcare landscape?

## Methods

### Study Design

This qualitative study adopted a constructivist approach acknowledging the researcher’s role in shaping and interpreting data [42]. To explore professional identity formation, we employed the concept of “navigating tensions” as it reflects the challenges pharmacists face, like balancing competing demands such as reconciling traditional dispensing duties with emerging clinical responsibilities. The theoretical framework of Boundary Crossing informed our data analysis, enabling us to understand how pharmacists navigate the evolving boundaries between these roles and develop their clinical identity within the context of comprehensive medication management.

### Study Setting and Participants

Clinical pharmacists across South Africa’s public sector with a postgraduate degree in clinical pharmacy and who practiced at least two hours per workday in public healthcare wards were eligible for participation in the study. This eligibility criterion reflects the current landscape of clinical pharmacy in South Africa.

We contacted all members of the South African Society of Clinical Pharmacists (SASOCP) who were known to practice in hospitals offering clinical pharmacy services. All clinical pharmacists meeting the criteria and consenting to participate were contacted for interview scheduling. For this study, “clinical pharmacist” refers to individuals with a Master of Pharmacy (M.Pharm) in Clinical Pharmacy.

South Africa’s public healthcare system includes approximately 628 registered pharmacies within public institutions [43]. Currently, South Africa has a total of 18,429 registered pharmacists [44], however, there is no official workforce data documenting how many pharmacists are employed in the public sector, nor how many work in clinical pharmacy roles. Clinical pharmacy is not formally recognized as a specialization, resulting in a lack of dedicated clinical pharmacy positions in the public sector. Currently, only 19 pharmacists are known to provide clinical pharmacy services in public hospitals, based on identification through SASOCP (South African Society of Clinical Pharmacists). This study includes the entire identified population of clinical pharmacists working in hospitals where clinical pharmacy services are part of their daily responsibilities.

### Data Collection

In November 2022, we conducted semi-structured interviews guided by the literature on professional identity and boundary crossing [13,35,38,45]. We focused on understanding how clinical pharmacists navigate the transition from dispensing to clinical roles, investigating the challenges they face in professional identity formation and interprofessional collaboration. The interview guide was iteratively revised by the research team and piloted with volunteers not involved in the study and can be found in the online Electronic Supplementary Material (Supplement 1). Prior to online interviews, participants received consent forms. Interviews began with introductions, consent restatement, and an opportunity for participant questions.

### Data Analysis

Thematic analysis, involving open and selective coding, was conducted using ATLAS.ti50. LC and AW iteratively read and coded all transcripts. Initially, descriptive codes were developed inductively, allowing the researchers to identify patterns and construct findings from the data. To deepen our understanding of boundary experiences and their impact on professional identity, we employed axial coding, mapping relationships between boundary experiences, participants’ response strategies, and their influence on professional identity formation.

Boundary experiences were classified as “temporary disruptions” in learning processes caused by differences encountered across and within contexts [35]. For example, such experiences were identified when clinical pharmacists faced internal conflicts about their evolving roles or when they encountered challenges in interactions with other healthcare professionals. The analysis involved both inductive and deductive approaches. To guide the analysis, a coding framework was developed based on the professional identity formation and boundary crossing literature [13,35,38,45]. The framework helped us understand pharmacists’ experiences and how they navigated these disruptions in the light of the broader concepts of interprofessional collaboration and identity formation. This was combined with open coding of other factors considered relevant to the research question. For example, we identified codes representing instances where pharmacists navigated internal challenges regarding their clinical roles, managed interactions with other healthcare professionals, and engaged in experiences that influenced their professional identity formation. We developed sub-themes inductively by grouping related codes, which were added and refined iteratively until they captured the full range of participants’ experiences [46]. This iterative process, including revisiting transcripts to refine these sub-themes, ensured that the coding scheme was responsive to participants’ evolving narratives.

We interviewed 12 out of the 19 identified clinical pharmacists working in the public sector, representing the maximum available sample of professionals who met the study’s eligibility criteria. As data collection progressed, recurring themes consistently emerged across participants, and the final interviews did not generate new codes, indicating data sufficiency [47]. This confirmed that information power was established, in line with Malterud et al. [16], as the sample size was sufficient to address the study’s objectives while capturing a diverse range of experiences [48].

To ensure coding trustworthiness and consistency, LC and AW independently coded a subset of transcripts. Consensus on the coding framework was achieved through ongoing discussions (LC, AW, MS, CW).

### Reflexivity and Rigour

The research team comprised four clinical pharmacists, a psychologist and educationalist, a psychologist, and a medical doctor, offering diverse expertise in medical education, qualitative research, and clinical pharmacy. LC, the interviewing pharmacist, also teaches in the M. Pharm program, providing valuable insight into interpreting participants’ experiences while acknowledging her potential influence. Following the constructivist paradigm of considering pharmacist experiences and researcher-participant interactions [49], LC remained mindful of this dynamic during data collection, using her experiences as a lens for understanding rather than shaping responses. All coding was discussed with a team member, and final themes were confirmed by the team.

This research was approved by the Sefako Makgatho Health Sciences University (SMUREC/P/232/2020:PG). Informed consent included explaining voluntariness, confidentiality, and reporting of the findings. No incentives were offered.

## Results

We invited all 19 clinical pharmacists working in the public healthcare sector to participate in the study, and 12 (nine female, three male) agreed to be interviewed. Participants were aged 35–54, held a Master’s degree in Clinical Pharmacy, and had an average of 17 years (range: 10–33 years) of pharmacy experience and 5 years (range: 5 months–12 years) of clinical or ward-based experience. By including a significant proportion of clinical pharmacists in the public sector, we captured a diverse range of experiences—from those actively engaged in direct patient care to those balancing clinical and dispensing duties, thus providing comprehensive insights into the profession. Interviews lasted between 40 and 90 minutes.

Interviews revealed participants’ experiences navigating boundaries related to their evolving role. These boundaries included: (a) Intrapersonal boundaries that emerged as pharmacists reconciled their traditional dispensing pharmacist role with their evolving clinical pharmacist role, and (b) Interpersonal boundaries that surfaced as pharmacists interacted with other healthcare professionals, negotiating their role within the healthcare team. Participants also described experiences that transcended these boundaries. These experiences involved navigating a complex interplay between their current work environment/practice setting, their professional values, and their past experiences. Participants described how their boundary experiences, shaped by both intra- and interpersonal factors, collectively influenced their professional identity formation within the complex healthcare environment. Our analysis yielded three key themes: [1] Bridging the gap within: Pharmacists navigate intrapersonal boundaries for clinical identity formation, [2] Bridging the gap between: Pharmacists navigate interprofessional boundaries for collaboration and identity formation, and [3] Building bridges: Pharmacists employ strategies to promote collaboration and recognition.

The results are illustrated with embedded quotes. Additional quotes (Q) for each theme are given in Supplement 2.

### Bridging the Gap Within: Pharmacists Navigate Intrapersonal Boundaries for Clinical Identity Formation

This theme reflects the complex internal experiences of clinical pharmacists transitioning from their traditional dispensing role to clinical practice in a complex healthcare environment. It explores how they navigate the intrapersonal tension between their established pharmacist identity and the formation of a new clinical identity. By focusing on challenges associated with role ambiguity and strategies employed to reconcile these competing identities, this theme provides insights into the personal transformation involved in developing a strong clinical pharmacist identity.

#### Challenges in defining the clinical pharmacist role and overcoming constraints

A key challenge was the lack of clearly defined clinical pharmacist positions, exacerbated by limited resources and time constraints, impacting pharmacists’ ability to develop their clinical roles. This ambiguity, combined with a demanding workload, made it difficult to create their role and establish a professional identity distinct from traditional dispensing pharmacists.

*“That is the main difference, I am now part of the management of the patient right from the beginning to the end. Where a community or dispensing pharmacist just needs to dispense the ‘take home’ medication. But clinical pharmacists have the full background of patients, that is why it’s such an exciting area of practice…but requires a clear role. (Participant 10)”*

*“The lack of support from management makes it difficult to carve out dedicated time and create a niche for ourselves. (Participant 7)”*

To embrace their expanded roles, pharmacists needed time and support to develop the necessary knowledge, skills, and confidence. This highlights the tension at the boundary, reflecting their struggle to reconcile their existing identity with evolving clinical responsibilities. Overcoming initial hesitations about their expanded role was crucial, for pharmacists to fully embrace the broader clinical perspective (essential for internal boundary navigation). This involved a shift in thinking processes from a dispensing-focused mindset to a patient-centered, clinical approach. This shift required acquiring new knowledge and skills (Q1, Q2), such as comprehensive medication management and interprofessional collaboration. Additionally, workload pressures due to understaffing and time constraints (Q3) necessitate a high degree of adaptability and resourcefulness (Q4).

#### Building Confidence and Identity: Overcoming Challenges and Leveraging Opportunities

Despite the challenges outlined above and resource limitations, participants demonstrated resilience, employing various strategies to actively shape their identity as clinical pharmacists.

A passion for continuous learning and patient care serves as a powerful driving force (Q5), empowering them to navigate challenges. This intrinsic motivation, coupled with their acquired skills, facilitated the formation of a strong clinical identity, even in the face of challenges. Strategies like continuous learning helped bridge internal boundaries, establishing pharmacists in their more dynamic and comprehensive roles. As they gained confidence and recognition within the team, their ability to assert their value strengthened, allowing them to contribute more effectively to patient care.

One participant reflected on this transformation, stating,

*“Being a clinical pharmacist has changed me… I’m seeing that now when I communicate with the doctors, their level of resistance towards me is really low and I feel a sense of assuredness in my role. I think I have grown and have the ability to effectively collaborate… They are able to listen and accept the role of the clinical pharmacist after working with them for a long time. (Participant 3)”*

This sense of assuredness and professional growth highlights how building confidence was facilitated by the pharmacists’ efforts to establish their place within the clinical team, such as advocating for dedicated positions and actively developing expertise (Q1, Q2). Advocating for dedicated positions allowed pharmacists to actively shape their professional environment, reinforcing their sense of agency and affirming their professional worth. This newfound confidence, stemming from advocacy and expertise development, empowered them to navigate internal tensions associated with their evolving roles. Positive feedback, encouragement, and mentorship from managers, established professionals, and networking opportunities can all validate pharmacists’ contributions, bolster confidence, and provide guidance for growth (Q6). Universities also play a key role by offering ongoing support, resources, and mentorship.

*“The university, most specially the lecturers and the professors that are encouraging us and motivating us to do more and supporting us with materials that we can use to better ourselves as clinical pharmacists in the setting. (Participant 3)”*

These experiences, in both formal and informal settings, help pharmacists navigate internal tensions associated with their evolving roles and shape their professional identity. While intrinsic motivation and a passion for patient care drive pharmacists to embrace their evolving roles, mentorship and external encouragement provide validation and opportunities to expand their clinical involvement, as highlighted in Quotes 5 and 6. By reconciling identities, pharmacists were able to develop a stronger, more confident clinical identity, potentially leading to a more cohesive approach to patient care.

### Bridging the gap between: Pharmacists navigate interprofessional boundaries for collaboration and identity formation

This theme reflects the interprofessional boundaries clinical pharmacists navigate while collaborating with other healthcare professionals. It focuses on the challenges they face in asserting their expertise, integrating into hierarchical teams, and overcoming communication gaps. These experiences influence their professional identity formation by highlighting issues like hesitation to intervene, unclear role expectations, and feelings of being undervalued due to systemic limitations. Such interprofessional tensions hinder pharmacists from fully establishing their roles and contributing effectively to patient care.

#### Interprofessional Boundary Negotiation: Challenges in Collaboration

Clinical pharmacists faced various challenges in navigating boundaries with other healthcare professionals. Interprofessional hierarchies and unclear role expectations contributed to hesitation in asserting their expertise and created friction within the team.

Pharmacists were often uncertain about their role and responsibilities, leading to reluctance to intervene when necessary.

One participant described their hesitation to speak up during medical rounds, stating,

*“Spotting a potential drug interaction during rounds, I hesitated to speak to the physician, worried about overstepping. Past experiences of doctors feeling micromanaged, like ‘I’m looking over their shoulder’ made me cautious. But the risk was high, so I raised it. He was initially dismissive, but the doctor appreciated my explanation and evidence and revised the patient’s plan. (Participant 9)”*

This quote highlights how hierarchical structures, past experiences and unclear role expectations reinforced pharmacists’ hesitancy, making it difficult for them to assert their expertise. This internal conflict, combined with external pressures to adhere to established hierarchies, further complicated their ability to confidently contribute to patient care. Pharmacists expressed confusion about their specific responsibilities (Q7) and felt only marginally integrated, often resulting in friction within the team. Hierarchical structures and misunderstandings within the team about the clinical pharmacist’s role further hindered their ability to fully integrate into interprofessional workflows.

Quote 8 illustrates this challenge, where a physician expressed surprise at a detailed medication plan proposed by the clinical pharmacist, highlighting the persistence of traditional hierarchies that limit the integration of clinical pharmacists. This interprofessional boundary, shaped by role ambiguity and resistance to shared decision-making, often resulted in team friction and potentially suboptimal patient care.

Beyond physician-pharmacist interactions, clinical pharmacists also encountered challenges in collaborating with dispensing pharmacists due to different priorities and communication gaps.

*“Calling pharmacy to check on a drug shortage, I felt like I was intruding. They seemed annoyed, asking why I couldn’t just use the usual substitute. It highlighted the siloed thinking– they see dispensing, we see the bigger picture. Makes it tough to collaborate for the best patient care. (Participant 12)”*

Dispensing pharmacists’ focus on immediate medication dispensing hinders clinical pharmacists’ efforts to gain a broader understanding of patient medication needs.

Some boundary experiences that shaped clinical pharmacists’ professional identity occurred within the context of specific limitations, such as the absence of official clinical pharmacy positions and limited support from colleagues, creating uncertainty and a sense of being undervalued, hindering their efforts to establish themselves (Q9).

Clinical pharmacists faced challenges in asserting expertise, gaining recognition, and overcoming established workflows that hampered integration of their role. These examples illustrate the boundaries encountered (interprofessional tensions) and challenges faced in navigating them effectively.

### Building bridges: Pharmacists employ strategies to promote collaboration and recognition

To overcome the intra- and interpersonal challenges (detailed in the previous themes) and to further develop their clinical identity, participants employed various proactive strategies to foster collaboration and achieve recognition within the healthcare team. These strategies were crucial not only for asserting their clinical roles but also for integrating more fully into the interprofessional team, thereby reinforcing their professional identity and enhancing their contribution to patient care.

**Advocating for Support and Resources:** By advocating for increased support and resources, such as dedicated staff or protected time for clinical activities (Q10), pharmacists attempted to create an environment that facilitated their contributions to patient care. These efforts were driven by the recognition that resource constraints hindered their ability to fully demonstrate their value and establish their roles within the healthcare team. By actively addressing these resource-driven boundaries, pharmacists not only wish to enhance their clinical practice but also seek to strengthen collaboration with other healthcare professionals, solidifying their position as integral members of the healthcare team.

**Building Trust and Enhancing Collaboration:** Overcoming challenges such as hesitation to intervene, as illustrated above, and advocating for their expertise (Q10) helped pharmacists build trust and improve collaboration with other healthcare professionals. As pharmacists became more confident in asserting their clinical role, they navigated interprofessional interactions with increased assertiveness and resilience, ultimately strengthening their role within the team.

**Demonstrating Value through Collaboration:** Participants voiced how actively participating in patient care alongside other healthcare professionals allows them to showcase their expertise. This expertise further facilitates a transition from dispensing to applying a more comprehensive clinical approach. Pharmacists who actively developed expertise felt better positioned to demonstrate their value to the team and contribute more effectively to patient care.

This collaborative approach can help build trust and recognition from colleagues, reducing interpersonal boundaries and establishing their role within the team.

*“Prior to my Masters, dispensing medication was a routine task, there’s not much you query. Now, as a clinical pharmacist, I’m a more critical thinker. I analyze prescriptions within the context of the patient’s full medical history, providing comprehensive care. Actively collaborating with colleagues on patient care demonstrates the value we bring to the team. (Participant 7)”*

**Taking Initiative and Overcoming Systemic Barriers:** By taking initiative beyond direct patient care, such as developing clinical guidelines and engaging in quality improvement projects participants showcase their broader capabilities and value to the healthcare system. This proactive approach/boundary crossing strategy strengthens their position within the team and contributes to the formation of their clinical pharmacist identity.

One participant described this proactive approach, stating,

*“The workload is overwhelming, and there aren’t enough hours in the day to work closely with colleagues. But I’ve started working on ward clinical guidelines and hospital quality improvement projects to make sure we can still improve patient care, even with the time constraints. (Participant 2)”*

These strategies highlight how pharmacists actively work to bridge the gaps and establish themselves within the team. However, the results also identified systemic barriers such as limited time, lack of support from management and workload pressures due to understaffing (Q3, Q4). The paper-based filing system adds to these challenges (Q11). These constraints create difficulties in balancing workload, collaborating effectively, and demonstrating their new role’s full potential. However, their desire for dedicated clinical pharmacist positions, along with their active efforts to bridge boundaries (Q12), reflects a strong commitment to overcoming challenges and establishing their role within the healthcare team, ultimately contributing to their professional identity formation.

## Discussion

This study explored how clinical pharmacists develop their professional identity within the complex South African healthcare landscape. Our findings reveal the intricate interplay between internal challenges—such as reconciling pharmacists’ traditional dispensing roles with clinical responsibilities—and external factors like navigating hierarchical structures and unclear role expectations within the healthcare team. This dynamic interaction between interpersonal and intrapersonal boundaries is essential in shaping professional identity. This study’s findings resonate with the broader literature on professional identity formation, which describes identity development as a process of negotiation between personal values and external professional expectations [29,31]. Our study contributes to a deeper understanding of the multifaceted factors influencing clinical pharmacists’ professional identity formation.

The South African healthcare context, characterized by limited resources [17,18], understaffing, and a lack of clearly defined clinical pharmacist positions [2,11], significantly influenced the experiences of participants in this study. These systemic challenges create additional obstacles to identity formation and professional development. Literature confirms that role ambiguity and resource limitations are common barriers in under-resourced settings [14]. However, our findings show that, unlike the structured career pathways in high-income settings, the lack of formal roles in South Africa compels pharmacists to actively shape their professional space [29].

Our findings showed that internal challenges such as role ambiguity, self-doubt, and reconciling competing identities are pivotal in shaping clinical pharmacists’ professional identity. Navigating these intrapersonal boundaries requires adaptability, resilience, and the acquisition of specialized skills, aligning with research that underscores internal conflicts as essential for identity formation [13,31,39]. Similar to findings in the health professions education (HPE) literature [31], these internal struggles, compounded by unclear career trajectories, challenge pharmacists in navigating evolving roles confidently. This process reflects Cruess et al.’s (2014) view of identity as an ongoing negotiation between internal self-concept and external expectations. By crossing the boundary between traditional dispensing roles and expanding clinical responsibilities, pharmacists in our study fostered a stronger clinical identity. Intrinsic motivation, continuous learning, and mentorship were key in overcoming challenges and building confidence, echoing Wald’s (2015) findings on mentorship’s critical role in helping professionals overcome self-doubt and build professional confidence. Additionally, our findings align with previous research on the value of intrinsic motivation, continuous learning [13], and mentorship as a foundation for identity formation [50]. In our study, reconciling these evolving identities further demonstrated pharmacists’ capacity to navigate internal tensions and embrace their expanded clinical roles.

Interpersonal boundaries, including misunderstandings about their roles, hierarchical structures, and communication gaps with other healthcare professionals, also influence professional identity formation [13]. However, pharmacists demonstrate resilience by advocating for their expertise, building trust, and actively participating in patient care. This aligns with Elvey et al. (2013), who found that role ambiguity and hierarchies often hinder pharmacists’ integration into interprofessional teams. Other studies echo the importance of applying theoretical knowledge in practice to navigate challenges and foster professional identity formation [50,51]. These findings reflect Wenger’s (1998) Community of Practice framework, emphasizing how active participation in professional communities fosters identity formation. Clinical pharmacists actively enacting their roles bears learning potential in itself [50], an essential skill for navigating challenges and establishing their roles within the healthcare system. Addressing interpersonal boundaries, such as navigating conflicting role models and expectations, remains essential for supporting professional identity formation [13].

The interplay between intrapersonal and interpersonal boundaries was evident in pharmacists’ experiences, with the lack of dedicated clinical positions central to many challenges. Limited resources and insufficient management support—tied to the absence of formal roles—erode self-confidence (intrapersonal) and make it harder for pharmacists to advocate for their expertise within the healthcare team (interpersonal) [35]. Without clear positions, pharmacists often face role ambiguity, undermining their ability to assert their place in the team, consistent with Hazen et al, (2018), who identified similar challenges of role ambiguity, affecting pharmacists’ integration into multidisciplinary teams. Conversely, building trust and overcoming resistance from colleagues (interpersonal) can boost self-confidence and foster a sense of belonging (intrapersonal) [7]. Our previous study emphasizes the importance of a clear understanding of clinical pharmacists’ roles by other healthcare professionals [7], which can create a positive work environment, enabling pharmacists to advocate for their expertise (interpersonal), increasing self-efficacy (intrapersonal), and strengthening their professional identity. Positive feedback and recognition from colleagues could further strengthen self-efficacy (intrapersonal), empowering pharmacists to take on new challenges and assert their roles more confidently (interpersonal), aligning with Bandura’s theory of self-efficacy [52]. This theory, widely applied in HPE, underscores how positive reinforcement fosters the confidence professionals need to assert their evolving roles [52].

Beyond the interplay of intrapersonal and interpersonal boundaries, boundary crossing competence is key to understanding the multifaceted nature of clinical pharmacists’ professional identity formation. While our data did not explicitly reveal the four learning mechanisms involved in boundary crossing (identification, reflection, coordination, and transformation), these processes may have implicitly shaped participants’ professional development. Akkerman & Bakker (2011) emphasize that boundary crossing competence, particularly in interprofessional contexts, is essential for integrating diverse perspectives and fostering professional growth. Essential elements for developing this skill include adaptability, resilience, flexibility, and the ability to integrate new knowledge [13,39]. These qualities enable pharmacists to navigate the complex, evolving demands of their roles, enhancing adaptability and fostering professional growth [41]. By developing boundary crossing competence—which involves interacting with individuals from different practices, integrating diverse perspectives, and engaging in personal development [41]—pharmacists can collaborate effectively, adapt to changing roles [8,12], and integrate new ideas into their practice [41,52]. This aligns with Hazen et al. (2018), who found that interprofessional boundary crossing helps overcome role ambiguity and build new professional identities. For instance, pharmacists may collaborate with researchers to develop new treatment regimens [13] or with policymakers to advocate for healthcare policy changes [7]. Developing boundary crossing competence requires clinical pharmacists to intentionally seek opportunities for interprofessional collaboration and learning from others [41]. Engaging in reflective practice is also crucial for exploring personal assumptions and gaining a deeper understanding of their identity and expertise [54], while mentorship and coaching could provide guidance in navigating boundary-crossing challenges [53]. Developing this competence allows clinical pharmacists to enhance their adaptability, and resilience, and to contribute to a more innovative and patient-centered healthcare system. As Wenger (1998) noted, communities of practice- where continuous learning and collaboration are central- play a significant role in fostering professional identity formation through boundary crossing.

Studies in HPE have similarly noted the value of boundary-crossing competence in fostering adaptability and enhancing professionals’ ability to collaborate effectively across disciplines [41]. Learning within communities of practice is essential for acquiring these skills [37]. Clinical pharmacists with strong boundary crossing competence can: 1) **Reconcile competing identities** by navigating internal tensions associated with their evolving roles to build a cohesive professional identity [54]; 2) **Build trust and collaborate effectively** by understanding others’ perspectives and seeking opportunities for collaboration; they can foster stronger relationships and improve teamwork [7,12,55,56]; 3) **Potentially overcome resistance from colleagues** through effective communication, negotiation, and conflict resolution skills. Clinical pharmacists could address misunderstandings and build trust with colleagues who may initially resist their involvement [7,55]; and 4) **Develop a strong sense of self-efficacy** by successfully navigating challenges and demonstrating competence, thereby enhancing self-belief and motivation [11,52].

### Recommendations

This study highlights critical areas for addressing the challenges clinical pharmacists face in LMICs, emphasizing that systemic reforms are foundational for fully integrating them into healthcare teams. Building on our previous work [12], we emphasize that without national policies establishing structured clinical roles- with clear responsibilities, standardized practice guidelines, protected clinical time, and formal recognition within multidisciplinary teams- pharmacists cannot be effectively integrated. Additionally, integrating clinical pharmacists into health policy frameworks is necessary to define their roles and provide the resources and infrastructure needed to align their practice with national health priorities.

However, even in the absence of formal positions and as systemic changes are pursued, addressing role ambiguity, limited resources, and mentorship gaps is critical to support professional identity formation and optimize pharmacists’ contributions [30]. To complement systemic changes, we propose the following measures: **Promoting reflective practice and continuous learning** for pharmacists navigating expanded roles [13]. Structured reflection and effective interprofessional communication are key for building confidence, enhancing collaboration, and improving patient care [13,39,50]. **Establishing communities of practice** [35,37] to support peer learning and mentorship, reduce professional isolation, and foster boundary-crossing competence [53]. **Expanding clinical pharmacy education programs and mentorship opportunities** to develop competencies needed for evolving roles. Programs should focus on fostering self-efficacy, building confidence, and enhancing adaptability. Developing soft skills like boundary-crossing competence and flexibility equips pharmacists to effectively navigate multidisciplinary environments. Future research could examine the long-term impact of professional identity formation on clinical outcomes and explore how communities of practice contribute to resilience and professional growth in LMICs. Longitudinal studies across diverse healthcare systems are needed to inform policy and practice.

### Limitations

The sample size was relatively small, reflecting the limited number of ward-based clinical pharmacists in South Africa’s public healthcare sector. Despite this, the sample effectively represents the demographics of existing practitioners. The experiences of pharmacists in the private sector may differ, necessitating further research. Our findings offer valuable insights for LMICs facing similar systemic challenges. While the mechanisms we found could be applicable in other contexts, we advise keeping the context in mind.

## Conclusion

The interplay between intrapersonal and interpersonal boundaries shapes clinical pharmacists’ professional identity in South Africa’s public healthcare system. Systemic challenges, such as the lack of official positions and limited resources, exacerbate these boundaries, hindering pharmacists’ potential. Developing boundary-crossing competence is key to navigating these challenges, helping pharmacists adapt to evolving roles and build confidence. Promoting interprofessional collaboration and establishing communities of practice supports continuous learning, reflective practice, and mentorship, further facilitating identity formation. By adopting these strategies, the contribution of pharmacists to patient care in LMICs can be enhanced and healthcare outcomes can be improved.

## Additional Files

The additional files for this article can be found as follows:

10.5334/pme.1597.s1 Supplement 1. Semi-structured Interview Guide.

10.5334/pme.1597.s2 Supplement 2. Participant quotes supporting Theme 1-3.

## Author Information

Lucille Crafford’s educational research interests include motivation and professional identity development in health professions education.
