# Supplementary material for: Navigating Boundaries: How Pharmacists Develop Their Clinical Identity in a Complex Multidisciplinary Healthcare Setting
Source: Perspect Med Educ. 2025 May 7;14(1):230–42. doi: 10.5334/pme.1597 (PMC12063577; doi:10.5334/pme.1597)
Supplement: Supplement 1. — Semi-structured Interview Guide. [file pme-14-1-1597-s1.pdf]

Semi-structured Interview Guide

1. Describe a work-week in your current job/employment position.
2. Describe the two hours or more in your week, where you function as a clinical pharmacist in a public sector institution.
3. What were your expectations of the function/role of a clinical pharmacist when you first started? (What did you hope for?)
4. What were the things you thought would be easy or difficult in practice?
5. Did or do you have any doubts regarding your role as a clinical pharmacist?
6. What is for you essential to your professional identity as a clinical pharmacist in a public healthcare institution?
7. What is your role in the wards you practice in?
8. What is your role in the hospital pharmacy (if applicable)?
9. Describe the difference in your way of work (and your way of acting) as a clinical pharmacist versus a community/dispensing pharmacist.
10. How do you introduce yourself to others?
11. What do you believe are the factors that have shaped your professional identity to this point?
12. Describe an interaction that you had in the past week that stood out with either a nurse or a doctor while following a patient in the ward setting (while delivering clinical pharmacy-related services).
13. What did you need from the doctor/nurse to successfully fulfil your function as clinical pharmacist?
14. How did the recent interaction with the doctor/nurse contribute to your professional identity development?
15. Can you identify other activities that you feel contribute to your professional identity development (as clinical pharmacist)?
16. What do you like best/least in your role as clinical pharmacist?
17. What do you find easy/hard in your role as clinical pharmacist?
18. How did your performance develop during the period that you worked in the general practice?
19. What is for you the added value of your work in hospital practice?
20. Which qualities are required to function effectively as a clinical pharmacist (in the hospital)?
21. How do you think the profession of clinical pharmacy will develop in the future?

22. What are the disadvantages and advantages of working as a clinical pharmacist in the public healthcare sector?

23. What do you perceive as facilitative and hindering conditions in the reported opportunities?
