# Supplementary material for: Navigating Boundaries: How Pharmacists Develop Their Clinical Identity in a Complex Multidisciplinary Healthcare Setting
Source: Perspect Med Educ. 2025 May 7;14(1):230–42. doi: 10.5334/pme.1597 (PMC12063577; doi:10.5334/pme.1597)
Supplement: Supplement 2. — Participant quotes supporting Theme 1-3. [file pme-14-1-1597-s2.pdf]

Supplement 2: **Participant quotes supporting Theme 1-3**

Quote	Quote # in text	Participant #
**Theme 1 on Intrapersonal Boundary Navigation for Clinical Identity Formation**		
**Challenges in Defining the Clinical Pharmacist Role**		
"The experience that I have working in the ICU has changed my thinking process. As a dispensing pharmacist you're not exposed to different uncommon scenarios, but as a clinical pharmacist, I've now become used to all of these new things. I am aware that there's almost always new guidelines and have to keep in mind that best practice is constantly changing. I think I have a broader perspective."	**1**	**6**
"...the level of knowledge I acquired with my Master's degree helps make those critical decisions regarding different medical conditions that patients present with….it also helps me handle the confusion around my role, especially with the lack of a dedicated position and the resource limitations we face every day."	**2**	**11**
"People have too much on their shoulders, especially because we are understaffed… everywhere is understaffed so people tend to get emotional when they don't see you in the mix running around."	**3**	**2**
"Think the hardest part is trying to juggle all that needs to be done and dividing yourself in 10 parts, sometimes it can be hectic"	**4**	**8**
**Building Confidence and Identity: Overcoming Challenges and Leveraging Opportunities**		
"I always wanted to be a clinical pharmacist… My passion for learning and improving patient care keeps me motivated"	**5**	**9**
"In recently attending an antimicrobial stewardship workshop at a university, I had simple conversations with Prof X and other clinical pharmacists, that I found very encouraging. They were just saying 'keep at it, you're doing well.' Prof was even saying he would like to invite me and my manager to Hospital X so he can maybe assist us in being able to spend more time in the wards, five hours is a lot of time in the wards but maybe even stay in the wards the whole day"	**6**	**6**
**Theme 2 on Interprofessional Collaboration and Identity Formation**		
**Interprofessional Boundary Negotiation: Challenges in Collaboration**		
"You get this type of saying that somebody will ask you as a clinical pharmacist 'but can't another pharmacist do your job?'... You know that it's not possible..."	**7**	**2**

"Before a patient I was following got discharged, I presented a medication plan tailored to the patient's specific needs to the doctor and nurses in the ward. While the nurses seemed receptive, the doctor expressed surprise that I'd prepared such a detailed plan. He clarified that medication decisions were typically made by them, the doctors. It felt like we missed a chance to collaborate, and like he didn't understand or appreciate my role in the team."	**8**	**1**
".. some people still don't know what clinical pharmacy is, some feel like we are just invading their space, or we are just there to tell them what to do"	**9**	**9**
**Theme 3 on Strategies for Promoting Collaboration and Recognition**		
"We need to advocate for more resources and support to better integrate with the team, as prioritizing time for clinical activities allows for more collaboration with colleagues on patient care."	**10**	**8**
"Working with patient records is a nightmare. It's like looking for a needle in a pile of papers...but we've been working on ways to improve the process so we can spend more time focusing on patient care."	**11**	**7**
"I'm hoping that we do get more clinical pharmacist positions so that the work will be more streamlined. But right now, we're finding ways to manage the workload and collaborate with the team on tasks like developing guidelines to show our essential role."	**12**	**10**
